# Lipoma in the Corpus Callosum Presenting with Epileptic Seizures Associated with Expanding Perifocal Edema: A Case Report and Literature Review

**DOI:** 10.1155/2015/520208

**Published:** 2015-05-13

**Authors:** Michiyasu Fuga, Toshihide Tanaka, Yohei Yamamoto, Yuzuru Hasegawa, Yuichi Murayama, Junko Takahashi-Fujigasaki

**Affiliations:** ^1^Department of Neurosurgery, Jikei University School of Medicine, Kashiwa Hospital, Chiba, Japan; ^2^Department of Neurosurgery, Jikei University School of Medicine, Tokyo, Japan; ^3^Division of Neuropathology, Jikei University School of Medicine, Tokyo, Japan

## Abstract

This report describes a rare case of a patient with lipoma presenting with epileptic seizures associated with expanding perifocal edema. The patient was a 48-year-old man who presented with loss of consciousness and convulsions. Magnetic resonance imaging (MRI) revealed a calcified mass in the corpus callosum with perifocal edema causing mass effect. An interhemispheric approach was used to biopsy the mass lesion. Histological examination revealed typical adipose cells, along with hamartomatous components. These components contained neurofilament and S-100-positive structures showing marked calcification. Fibrous cells immunoreactive for *α*-smooth muscle actin and epithelial membrane antigen proliferated with focal granulomatous inflammatory changes. MIB-1 index was approximately 5% in immature cells observed in granulomatous areas. We thus suspected a coexisting neoplastic component. The residual lesion persisted in a dormant state for 2 years following biopsy. Surgical resection of a lipoma is extremely difficult and potentially dangerous. However, in the present case, the lesion was accompanied by atypical, expanding, and perifocal edema. Surgical treatment was inevitable for the purpose of histological confirmation, considering differential diagnoses such as dermoid, epidermoid, and glioma. In the end, anticonvulsant therapy proved effective for controlling epileptic seizures.

## 1. Introduction

Intracranial lipomas are rare, accounting for 0.1–0.5% of all primary brain tumors. Intracranial lipomas are attributed to abnormal differentiation of persistent primitive meninx (mesenchymal origin), followed by transformation into mature adipose cells [1]. The lesion constitutes an inner level of the pia, arachnoid, and dura [2]. Lesions occur frequently at or near the midline, mostly in the pericallosal cistern. Other locations include the quadrigeminal plate, superior cerebellar peduncle, suprasellar cistern, cerebellopontine angle cistern, and sylvian cistern [1–4].

In general, intracranial lipomas are asymptomatic. Epileptic seizures are a common symptom and are sometimes refractory to anticonvulsant treatment [5–10].

Histological findings of intracranial lipoma have been described in detail in the literature [11, 12]. Budka described the histological findings containing extensive adipose cells along with lipomatous hamartomas, emphasized by the possible occurrence of muscle fibers, nerve cells, calcification, and bone formation [11].

Differential diagnosis for intracranial lipoma includes radiographically similar lesions such as dermoid, epidermoid, and gliomas, and differentiation from these pathologies is important for appropriate therapeutic planning.

Radical surgical resection is usually contraindicated because of the nature of the lesion and may result in high morbidity and mortality rates, given the vascularity and strong adhesion to surrounding tissue [2, 13]. However, surgery was considered inevitable for histological confirmation especially in case of the mixed intensity mass lesion associated with atypical expanding perifocal edema as in the present case.

## 2. Case Report

**Figure 1:**
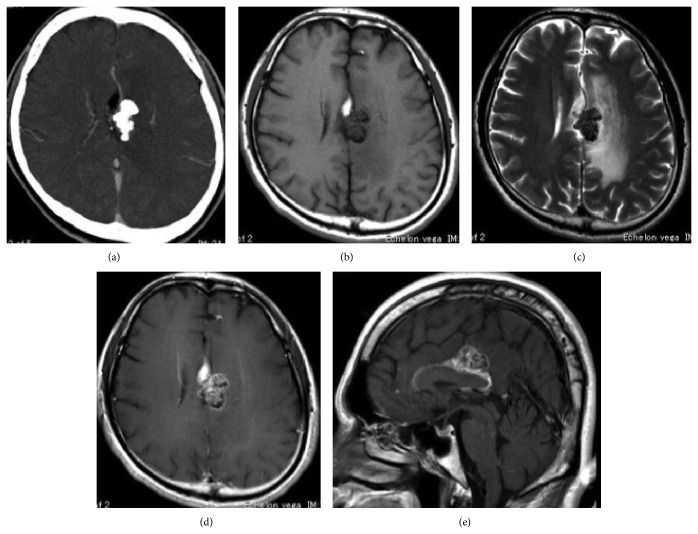
(a) Preoperative computed tomography (CT) showing calcified tumor in the interhemispheric fissure. (b, c) Preoperative magnetic resonance imaging (MRI) showing a lesion along the corpus callosum, appearing hyperintense on T1-weighted imaging (b) and isointense on T2-weighted imaging (c) associated with expanding perifocal edema. (d, e) The mass shows heterogeneous enhancement with gadolinium or axial (d) and sagittal images (e).

A 48-year-old man presented with loss of consciousness on two occasions and paresthesia of the right extremities. A calcified cystic lesion was noted in the corpus callosum on computed tomography (CT) (Figure 1(a)). Magnetic resonance imaging (MRI) showed a lesion with two components appearing hyper- and isointense on T1- and T2-weighted imaging with heterogeneous gadolinium enhancement (Figures 1(b)–1(e)).

**Figure 2:**
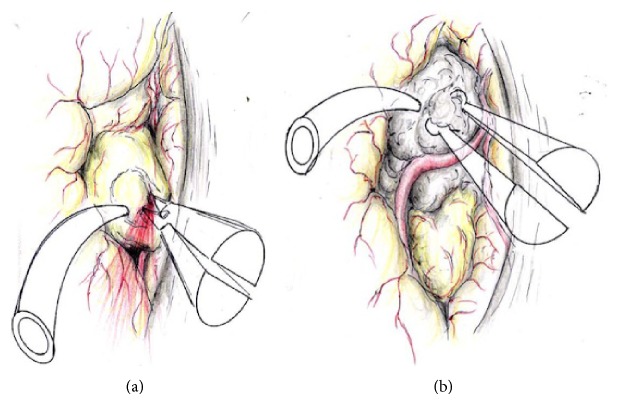
Schema of intraoperative findings. (a) Incision of the initially yellowish tumor (lipoma component) results in projectile bleeding. (b) The grayish tumor is exposed just behind the A3. Residual lipoma is observed posteriorly.

As the differential diagnoses included oligodendroglioma, metastasis, and teratoma, in addition to lipoma, the patient underwent left frontal craniotomy via an interhemispheric approach. The mass was located extra-axially and was not attached to the falx (Figure 2), consisting of two components corresponding to the MRI findings (Figures 2(a) and 2(b)). An elastic yellowish tumor was initially identified just above the corpus callosum and showed easy bleeding on incision. Macroscopic findings corresponded to lipoma. Following electrocauterization of the lipoma, a grayish tumor posterosuperior to the lipoma was identified just behind the pericallosal artery.

**Figure 3:**
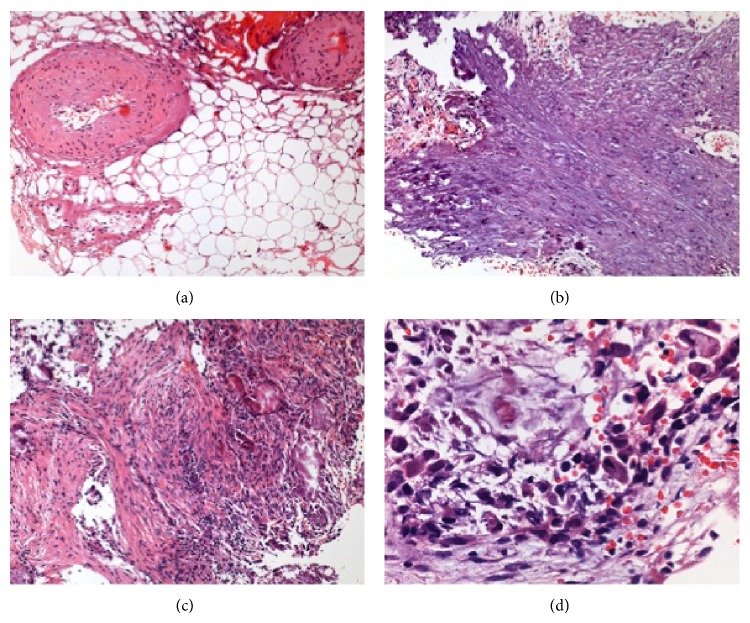
(a) Histological findings reveal mature adipose tissue with a small amount of collagen and thickened blood vessels from the yellowish tumor. (b, c) Greyish tumor shows marked calcification in most parts (b) and spindle-shaped cells in the sheet (c). Infiltration of mononuclear inflammatory cells along with immature cells showing hyperchromatic nuclei. Hematoxylin and eosin: (a–c) ×100; (d) ×400.

**Figure 4:**
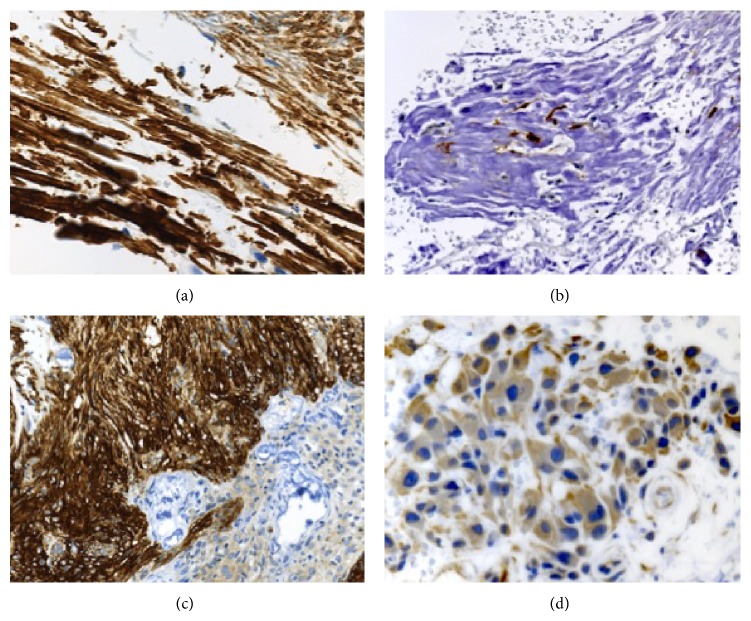
(a) Immunohistochemical findings showing strong positive staining for neurofilament (NF) in calcified areas. (b) Small foci in calcified areas are positive for S-100. (c) In noncalcified areas, spindle cells are positive for *α*-smooth muscle actin (SMA). (d) Immature cells with hyperchromatic nuclei along with infiltrating mononuclear inflammatory cells are weakly positive for vimentin. Hematoxylin and eosin: (a) ×400; (b) ×200; (c) ×200; (d) ×400.

Biopsied specimens were taken from three regions: the yellowish tumor, the grayish tumor, and brain parenchyma around the tumors. The yellowish tumor consisted of mature adipose tissue containing a small amount of collagen and thickened blood vessels (Figure 3(a)). Several small specimens from the grayish tumor displayed marked calcification in most parts (Figure 3(b)). In addition, granulomatous inflammatory reactions were seen in the noncalcified areas. Infiltration of mononuclear inflammatory cells was identified, along with proliferation of small vessels (Figure 3(c)). Immature cells with hyperchromatic nuclei were also present (Figure 3(d)). The calcified areas displayed selective, strong immunostaining for neurofilaments (NFs) (Figure 4(a)). Occasionally, fiber-like linear staining of NFs arranged in parallel was seen, and small, S-100-positive foci were found in the calcified areas (Figure 4(b)). In noncalcified areas of the specimens, spindle cells immunopositive for *α*-smooth muscle actin (SMA) were seen intersecting fascicles with deposition of collagen fibers (Figure 4(c)). Immunoreactivity for epithelial membrane antigen (EMA) was observed in these areas. Immature cells with hyperchromatic nuclei showed weak immunostaining for vimentin (Figure 4(d)). Immunohistochemical staining for desmin and glial fibrillary acidic protein yielded negative results. CD45- and CD68-positive cells were detected. The MIB-1 index was 5% in the most immature cells. Reactive gliosis was observed in brain parenchyma.

The postoperative course was uneventful. MRI at 3 years postoperatively revealed that the mass remained dormant without adjuvant therapy. As of 3 years postoperatively, the patient remains free of epileptic seizures with the aid of anticonvulsant (400 mg daily carbamazepine).

## 3. Discussion

Intracranial lipoma was originally described in 1856 by von Rokitansky and was considered a benign, slow-growing, and congenital hamartomatous condition [14]. These lesions account for only 0.1–0.5% of all primary brain tumors [1, 2, 6]. Truit described the pathogenesis of intracranial lipoma after reviewing data from 42 patients with 44 intracranial lipomas [1]. Lipoma results from the abnormal persistence and maldifferentiation of the embryonic meninx primitiva during the development of the subarachnoid cistern [1].

In general, lipomas are asymptomatic, and the association between epileptic seizures and intracranial lipoma remains controversial. When symptomatic, lipomas in the sylvian fissure or cortex, or associated with focal cortical dysplasia, usually present with epileptic seizures, presumably due to irritation of the cortex [5–10]. It is unclear whether or not the lipoma associated with expanding perifocal edema in the present case probably causing focal epileptic seizure due to interhemispheric disconnection or infiltration of the cingulate gyri as Gastaut et al. described [7]. More specifically, electroencephalographic findings in lipoma with epilepsy revealed seizure foci in some case reports [5, 8].

Surgery is unnecessary for stable or asymptomatic cases, since the risks for radical resection far outweigh any potential benefits [2, 13]. Even in cases when the lipoma is associated with epileptic seizures, surgery will not cure the seizures; therefore, antiepileptic medications are usually successful in controlling symptoms [6].

As described above since the radical resection was seldom attempted, very few reports have described histological findings of the intracranial lipoma [11, 12, 14, 15], although the precision of etiology of lipomas is not well understood. As in the present case, which revealed the calcified mass with expanding perifocal edema, biopsy is occasionally necessary for differential diagnosis from the neoplasms.

Rubinstein described that the intimate relationship of neuroglial to mesenchymal elements in intracranial lipomatous hamartoma possibly coexisted with muscle fibers, nerve cells, calcifications, and bone formation [12]. Histological features of intracranial lipoma reveal malformative characteristics with structured gliomesenchymal “mixed” tissue. In a review of 13 cases showing lipomatous hamartomas, Budka advocated that intracranial adipose tissue masses should be regarded as a true malformation and the term “lipoma” should be abandoned as incorrectly implicating a neoplastic character [11]. Gerber and Plotkin described immunohistological findings of negative results for SMA, HMB-45, and myogenin at autopsy [16]. Microscopically, a dense collagenous capsule is usually adhered to the adjacent brain. As in the present case, the anterior cerebral artery passed through the tumor and divided into pericallosal artery and callomarginal artery. The yellowish part was a lipoma with highly vascularity, and consisting of mature adipose tissue with variable amounts of collagen, blood vessels, and calcification [16, 17]. Accumulation of calcium has been found within the lipoma capsule, in the lipoma itself, and in the surrounding brain tissue [16]. The grayish part consisted of hamartomatous components including nerve fibers with marked dystrophic calcification and proliferation of smooth muscle-like spindle cells. The presence of Schwann-like cells was indicated by spotty S-100 positivity in the calcified areas, although detailed structures of the S-100-positive components were difficult to examine due to the marked calcification. Inflammatory reactions were present in these components, and immature cells seemed to appear concomitantly. The MIB-1 index of immature cells was approximately 5%, suggesting that the lesion was probably not a pure lipoma but the possibility of an admixed hamartomatous or neoplastic component. The residual lesion has remained dormant for 3 years, so these findings were considered nonneoplastic. Truit suggested that intracranial lipomas are neither hamartomas nor true neoplasms [1]. The etiology and pathophysiological significance of the densely cellular lesion resulting in degenerative granulation associated with lipoma could not be elucidated. Based on postoperative clinical course and histological findings, we concluded that the final diagnosis in the present case was the lipoma coexisted with not tumor but hamartomatous change.
